# MicroRNAs Involved in the Lipid Metabolism and Their Possible Implications for Atherosclerosis Development and Treatment

**DOI:** 10.1155/2014/275867

**Published:** 2014-04-24

**Authors:** Jan Novák, Julie Bienertová-Vašků, Tomáš Kára, Miroslav Novák

**Affiliations:** ^1^International Clinical Research Center, Department of Cardiovascular Diseases, St. Anne's University Hospital Brno, Pekarska 53, 656 91, Brno, Czech Republic; ^2^Department of Physiology, Faculty of Medicine, Masaryk University, Kamenice 5, Building A20, 625 00 Brno, Czech Republic; ^3^Department of Pathological Physiology, Faculty of Medicine, Masaryk University, Kamenice 5, Building A18, 625 00 Brno, Czech Republic

## Abstract

Hyperlipidemia is a well-accepted risk factor in the development of atherosclerosis. MicroRNAs (miRNAs), a novel class of posttranscriptional regulators of gene expression, are involved in a variety of biological and pathological processes, including the regulation of the lipid metabolism and atherosclerosis. As our knowledge of miRNAs expands, a new class of “circulating miRNAs” has recently been described. It includes miRNAs which may be found in various bodily fluids packaged in microvesicles/exosomes, or bound to specific transporting proteins. High-density lipoprotein (HDL) particles have been identified as one such carrier. As this class of miRNAs likely plays a role in intercellular communication, it may also contribute to the atherosclerosis development and progression. This review aims to provide a comprehensive explanation of the roles of distinct miRNAs involved in the regulation of the lipid metabolism. These microRNAs seem to be promising therapeutic agents, as documented in rodents and African green monkeys. The second part of the review focuses on circulating miRNAs and their involvement in the atherosclerosis, especially as their levels have been described as altered in patients with dyslipidemia/hyperlipidemia. Special emphasis is placed on miRNAs transported in a complex with HDL particles and on those which may be considered potential atherosclerosis biomarkers.

## 1. Introduction

Atherosclerosis is a multifactorial complex disease triggered and maintained by a low-level chronic inflammation of the arterial wall [1]. The onset of atherosclerosis includes a dysfunction of endothelial cells, caused by a variety of external stimuli (e.g., hypertension, reactive oxygen species, or modified low-density lipoprotein (LDL) cholesterol). The damaged endothelium consequently begins to express more adhesive molecules, for example, vascular cell adhesion molecule 1 (VCAM-1), leading to promoted adhesion and infiltration by immune system cells [1]. The further development of atherosclerosis is influenced by a variety of risk factors, of which hyperlipidemia is considered one of the most important [2]. Severe hyperlipidemia promotes the progression of atherosclerosis and its end-points, that is, myocardial infarction or stroke, as shown in patients with familial hypercholesterolemia who suffer a myocardial infarction or stroke at a very early age [3–5].

Key players in hyperlipidemia and atherogenesis development include LDL and high-density lipoprotein (HDL) cholesterol. LDL cholesterol may be considered a typical proatherogenic substance while HDL cholesterol is considered antiatherogenic [43, 44]. LDL particles are normally removed from circulation by LDL-receptors (LDL-R). In patients with hyperlipidemia, various molecular pathways involved in the lipid metabolism are affected, resulting in changes to circulating lipid levels. LDL-Rs are often downregulated in such patients, which leads to the prolonged circulation of LDL particles in the bloodstream and subsequently to LDL modification, most commonly oxidation (oxLDL) [1, 45, 46]. OxLDL molecules may be effectively removed from circulation by scavenger receptors (SR) located on macrophages; however, since this process is not controlled as well as LDL-R-mediated removal, it results in the formation of foam cells which are often identified in atherosclerotic lesions as promoting the development and formation of atherosclerotic plaques [47]. In order to reverse the formation of atherosclerotic plaques, HDL particles are capable of ensuring reverse cholesterol transport, that is, cholesterol transport from peripheral tissues (e.g., atherosclerotic plaques macrophages) back to the liver. The improvement of reverse cholesterol transport via HDL could thus be beneficial for patients suffering from hyperlipidemia and atherosclerosis [43].

The recognition of microRNAs (miRNAs, miRs), tiny noncoding RNA molecules known to be involved in the posttranscriptional regulation of gene expression, facilitates the further understanding of the atherosclerosis process [48–50]. miRNAs are transcribed from corresponding genes located within intergenic regions or embedded within the introns of known protein-coding genes using RNA polymerase II/III [51]. The transcribed molecule is called primary-miRNA (pri-miR). Subsequently, within the nucleus, pri-miR is cleaved with the enzyme Drosha to precursor miRNA (pre-miR) which is then transferred to cytoplasm via exportin-5/RanGTP. In the cytoplasm, the pre-miR is cleaved with nuclease Dicer, which results in the creation of a miRNA duplex consisting of a mature miRNA strand (miR) and a passenger miRNA strand (miR*) [51]. The mature miRNA is usually more stable and is then loaded into the RNA-induced silencing complex (RISC) with Argonaut (Ago) proteins. Within this complex, mature miRNA binds to the 3′-untranslated (3′UTR) region of its target mRNAs, which results either in target mRNA degradation (in case base complementarity is complete) or in a mRNA blockade and the inhibition of protein translation (in case complementarity is incomplete) [49, 51]. Although the miR* strand is commonly degraded, some miR* strands have been loaded into the RISC as in the case of mature miRNA strands, resulting in the inhibition of translation or degradation of other target mRNAs [9].

The posttranscriptional regulation of gene expression described above takes place within the cell (intracellularly). However, a novel class of recently described “circulating miRNAs” [52] may be found in the extracellular space [52, 53]. These miRNAs may influence and further our understanding of atherosclerosis to a great extent, as they were found to be both stable (which makes them viable future biomarkers [54]) and also functional in terms of facilitating the transport of miRNA from one cell to another, thus enabling intercellular communication [53].

This review aims to describe the roles of miRNAs participating in the lipid and lipoprotein metabolism, with special emphasis placed on their possible involvement in the development and progression of atherosclerosis. The roles of circulating miRNAs known to be involved in atherosclerosis will also be discussed briefly in order to provide the concluding rationale for the use of miRNAs as possible atherosclerosis diagnostic and therapeutic tools.

## 2. MicroRNAs Involved in the Lipid Metabolism 

Various molecules have previously been shown to play key regulatory roles in the lipid metabolism, including nuclear transcription factors: sterol regulatory element-binding protein (SREBP), liver X receptor (LXR), or farnesoid X receptor (FXR) [55]. SREBP, LXR, and FXR, along with other molecules including various miRNAs, are closely involved in the orchestration of the proper course of the lipid metabolism. This section is dedicated to providing an overview of the functions of miR-33, miR-122, miR-27a/b, and several other miRNAs (see Table 1) which were shown to be involved in this complex regulatory network.

### 2.1. MicroRNA-33 and MicroRNA-33*

In 2004, Rodriguez et al. identified miR-33, embedded within intron 16 of the* SREBP* gene [56]; however, it took several years of additional study to find out that, together with its host gene, miR-33 regulates the metabolism of cholesterol and fatty acids [7, 8, 11]. Two members of the miR-33 family–miR-33a and miR-33b—which differ in only two nucleotides and which are embedded in the introns of* SREBP-2* and* SREBP-1*, respectively, are present in humans [57]. Rodents, on the other hand, only have one* SREBP* and miR-33 form [57].

As mentioned in the introduction, during miRNA biogenesis, pre-miR-33 (as well as any other pre-miRs) is cleaved with Dicer, which leads to the creation of a miR-33 duplex composed of mature (miR-33) and passenger (miR-33*) miRNA strands. As shown by Goedeke et al. in 2013, miR-33 is one of the examples where both strands are loaded into RISC and consecutively execute their tightly related function, thereby affecting cholesterol metabolism [6–11], fatty acids metabolism [9, 58], and glucose metabolism/insulin signaling [9, 58, 59].

**Figure 1:**
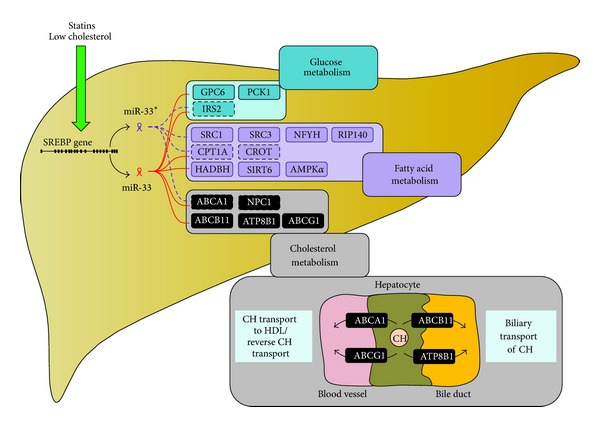
MicroRNA-33/33* effects. MicroRNA-33/33* are transcribed from the intron of the* SREBP* gene. Statins and low circulating cholesterol levels promote this process. Both miRNAs also target a variety of mRNAs involved in the glucose, fatty acid, and cholesterol metabolism. In the cholesterol metabolism, their targets include molecules involved both in cholesterol transport to HDL/reverse cholesterol transport and the transport of cholesterol to bile. CH: cholesterol; all other abbreviations are explained in the text.

Within the cholesterol metabolism, both miR-33 and miR-33* target ATP-binding cassette A1 (ABCA1) and Niemann-Pick disease C1 (NPC1), that is, molecules important for loading cholesterol into HDL particles and for cholesterol transport from the lysosomal compartment within the cell, respectively [6, 7, 9, 10]. MiR-33 itself was further shown to target ABCG1 in mice, which has a similar function to ABCA1, that is, enabling HDL formation and reverse cholesterol transport (e.g., cholesterol transport from atherosclerotic plaque macrophages back to the liver); however, this target was not confirmed in humans [6, 10]. Interestingly, both ABCA1 and ABCG1 are under the transcription control of LXR [10] and miR-33 thus provides the connecting link between SREBP-induced cholesterol synthesis and retention and LXR-mediated cholesterol efflux and reverse transport [55]. In addition to above described mechanisms, miR-33 targets ABCB11 and ATP8B1, both of which are important molecules in cholesterol efflux into biliary ducts; this targeting thus supports cholesterol retention caused by the upregulation of SREBP and miR-33 in hepatocytes [11]. This upregulation may be induced by extracellular signals including low circulating cholesterol levels or statin therapy [11] (Figure 1).

Confirmed targets for both miR-33 and miR-33* in the fatty acid metabolism include carnitine palmitoyltransferase 1A (CPT1A) and carnitine O-octaniltransferase (CROT) [8, 9, 58]. Furthermore, miR33 also targets hydroxyacyl-CoA-dehydrogenase (HADBH) [8, 58], sirtuin-6 (SIRT6), and AMP-activated protein kinase subunit-*α* (AMPK*α*) [58], while miR-33* targets steroid receptor coactivator 1 (SRC1), SRC3, nuclear transcription factor Y (NFYC), and receptor-interacting protein 140 (RIP140). By regulating all of these molecules posttranscriptionally, both miR-33 and miR-33* reduce fatty acid oxidation when upregulated and vice versa, that is, stimulating the process when downregulated [9, 58]. Needless to say, miR-33 also influences insulin signaling by targeting insulin receptor substrate 2 (IRS-2), thereby affecting both lipid and glucose metabolisms [58], with the glucose metabolism also being affected by glucose-6-phosphatase (G6PC) and phosphoenolpyruvate carboxykinase (PCK1) targeting [59].

According to the above-described roles of miR-33 and miR-33* in the cholesterol, fatty acids, and glucose metabolism, the therapeutic downregulation of their signaling may be beneficial for patients suffering from atherosclerosis, as it would result in an increase of HDL levels and in a decrease in fatty acid and glucose levels. This possibility will be examined in detail in the last section of this review.

### 2.2. MicroRNA-122

MiR-122 was identified in 2002 by Lagos-Quintana et al. to be liver-specific, accounting for over 70 % of liver miRNA content [60]. It is located within exon 2 of the known noncoding RNA gene* hcr* (gi: 51212) [56, 61]. This miRNA is currently believed to be essential for hepatitis C virus replication [62], hepatocellular carcinoma biogenesis [63], and—last but not least—for the functioning of the lipid metabolism [12–15, 64]. However, since its effects on the lipid metabolism were observed in studies using either antisense oligonucleotides (ASO) [12, 13, 64] or knock-out animals [14, 15]—both of which led to the inhibition of miR-122 function—it is still not precisely determined whether the effect of miR-122 on the lipid metabolism is direct or indirect [65].

The introduction of ASO into experimental animals leads to the creation of stable heteroduplexes, is reversible, and shows no signs of hepatotoxicity [64]. Studies using ASO to silence miR-122 showed that the introduction of anti-miR-122 leads to a decrease in plasmatic cholesterol levels in experimental animals [12, 13]. Moreover, Esau et al. observed that hepatic fatty acid oxidation is increased in anti-miR-122 animals, along with a decrease in both cholesterol and fatty acid synthesis [13]. Furthermore, the introduction of anti-miR-122 into a diet-induced obesity model led to the improvement of liver steatosis [13]. Microarray analysis and RT-PCR confirmation indicated that levels of an enormous number of mRNAs in the liver are changed following anti-miR-122 delivery. One of these mRNAs is SREBP, which may imply a connection between miR-122 and miR-33/33* functions [13].

A more prominent decrease in circulating levels of cholesterol and fatty acids was observed when miR-122a knockout animals were generated [14, 15]. On the other hand and contrary to results obtained in ASO studies, lipids were found to accumulate in the liver, resulting in hepatosteatosis and liver inflammation, which in turn led to fibrosis and the occurrence of spontaneous tumors resembling hepatocellular carcinomas [14, 15]. The reintroduction of miR-122 led to a significant improvement of steatosis while also suppressing tumorigenesis [14, 15]. Further studies are therefore needed in order to explain the differences observed between miR-122 silencing using ASO and miR-122 knockdown.

### 2.3. MicroRNA-27a/b

The effects of miR-27a on the lipid metabolism were extensively studied by Shirasaki et al. [16]. MiR-27a was thus identified as targeting RXR*α*, ABCA1, FASN, SREBP1, SREBP2, peroxisome proliferator-activated receptor *α* (PPAR*α*), and PPAR*γ* as well as ApoA1, ApoB100, and ApoE3 [16], all of which are molecules significant for the lipid metabolism and some of which were mentioned above. MiR-27b, having 27 predicted lipid metabolism-related targets, was identified as representing a “posttranscriptional miRNA hub”, that is a connection point, for the various parts of the lipid metabolism [18]. Of these targets, PPAR*γ*, angiopoietin-like 3 (ANGPTL3), N-deacetylase-N-sulfotransferase 1 (NDST1), and mitochondrial glycerol-3-phosphate acyltransferase (GPAM) were successfully validated [18]. PPAR*γ* and CCAAT/enhancer-binding protein *α* (C/EBP*α*) are both important for adipocyte terminal differentiation and their downregulation caused by miR-27b results in an adipocyte differentiation blockade [19]. An additional PPAR transcription factor, that is, PPAR*α*, which regulates the expression of, for example, ABCA1 or ABCG1 transporters, was also shown to be a putative target of miR-27b [17, 20]. Needless to say, miR-27b levels may be altered in atherosclerotic patients as they are sensitive to hypoxia and circulating lipid levels [19] and are upregulated in ApoE^−/−^ mice [18].

### 2.4. Additional MicroRNAs

**Figure 2:**
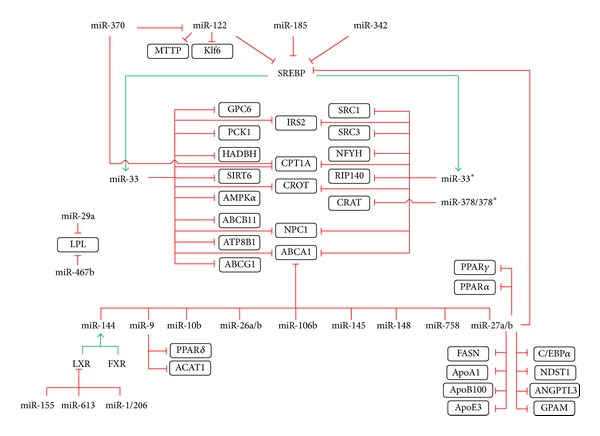
MicroRNAs involved in lipid metabolism. The figure depicts the complex regulation of lipid metabolism by distinct microRNAs. Individual microRNAs are shown together with their confirmed targets (in rectangles); red blunted arrows represent inhibition; green arrows represent stimulation. Figure summarizes the relationships described into greater detail in the text. The abbreviations are also explained in the text.

Except for the three miRNAs described above, a number of additional miRNAs is also involved in the lipid metabolism (Figure 2), though information about their effects is limited to a relatively small number of studies. MiR-370 was identified as affecting miR-122 expression, which in turn affects the lipid metabolism through miR-122 targets [21]. MiR-370 itself targets CPT1A, one of the miR-33/33*targets important for fatty acid oxidation [21]. Moreover, in patients with hyperlipidemia and coronary artery disease (CAD), levels of circulating miR-370 and miR-122 correlate positively with the severity of CAD [66], thus indicating that these two miRNAs may potentially be used as CAD biomarkers.

MiR-144 is another recently identified miRNA which affects the lipid metabolism [22, 23]. The promoter region of this miRNA includes two FXR-binding sites and indeed FXR drives miR-144 upregulation, resulting in a decrease in ABCA1 transporter, thus inhibiting HDL formation [22]. A similar effect may be observed in the case of LXR-activated macrophages and hepatocytes in high-fat diet-fed mice [23], indicating that both LXR and FXR control miR-144 expression and thus ABCA1 function. In both of the above mentioned studies, the silencing of miR-144 resulted in an increase in ABCA1 and HDL, that is, an effect similar to that of anti-miR-33 therapy [22, 23].

**Figure 3:**
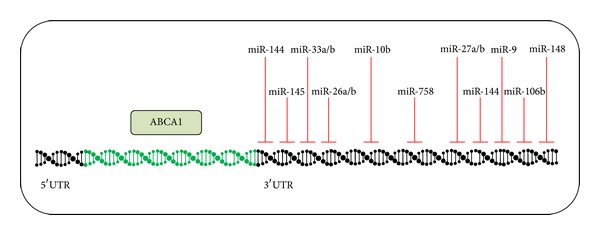
MicroRNAs targeting ABCA1 mRNA. This figure summarizes all currently validated miRNAs which target ABCA1 mRNA and are thus involved in cholesterol transport. The inhibition of these miRNAs holds great therapeutic potential in increasing circulating HDL cholesterol levels.

From a miRNA point of view, the ABCA1 transporter itself is a very attractive molecule (Figure 3): its 3′UTR is remarkably long-over 3 kb [67]—and holds miRNA-binding sites for miR-10b [68], miR-26 [69], miR-27 [16, 70], miR-33 [6, 7, 10], miR-106b [71], miR-144 [22, 23], miR-145 [70], miR-148 [70], and miR-758 [72]. All of these miRNAs clearly also have other targets (e.g., ABCG1 for miR-10b [68]), thus opening up space for extensive future research.

Additional miRNAs with corresponding targets involved in various parts of the lipid metabolism which may affect atherosclerosis development are summarized in Table 2.

## 3. Circulating MicroRNAs in Atherosclerosis

A very large number of microRNAs have been described as playing a role in the atherosclerosis process (for a recent review, see [50]). This section briefly discusses circulating miRNAs known to be involved in intercellular communication within atherosclerosis [73–76] and focuses specifically on circulating miRNAs transported by HDL or LDL particles [53, 77].

### 3.1. Circulating MicroRNAs

Unlike most of the above mentioned “tissue” or “intracellular” miRNAs, circulating miRNAs are present in extracellular space as well as in almost all bodily fluids: blood and its derivatives (i.e., serum and plasma) [52], cerebrospinal fluid, saliva, urine, breast milk and colostrum, bronchial lavage, peritoneal and pleural fluid, seminal fluid, and tears [78]. Since the addition of synthetic miRNAs directly into plasma leads to immediate degradation due to high RNAse plasma activity, the search for mechanisms by which circulating miRNAs are stabilized has been initiated [52]. As a result, several mechanisms for miRNA protection and transport were identified, including the packaging of miRNAs into exosomes [79], microvesicles [80], and apoptotic bodies [81] or the creation of stable complexes with Ago proteins [82] or lipoprotein particles [53].

The origin of circulating miRNAs varies according to particular miRNA species. While some miRNAs are released into circulation due to necrosis (e.g., miR-208 following myocardial infarction [83]) or apoptosis (e.g., miR-126 following epithelial cell apoptosis [81]), there is evidence of some miRNAs being actively secreted by cells [53, 79, 84]. The profiles of circulating miRNAs are thus not random [85] and reflect the physiological and pathological states of various tissues. Moreover, as bodily fluids are easily collectable, their utilization as disease biomarkers in the future is clearly a viable option [54].

### 3.2. Circulating miRNAs and Atherosclerosis

Circulating miRNA profiles have been shown to differ in patients suffering from atherosclerosis [77, 86, 87] and hyperlipidemia [66, 87]. It is thus very likely that these changes affect intercellular communication and contribute to the progression of atherosclerosis [88].

One of the first studies to point out the different expression of circulating miRNAs in the blood of CAD patients was conducted by Fichtlscherer et al. [86]. This study indicated that levels of endothelial enriched miRNAs, that is, miR-126, miR-17, and miR-92a, as well as inflammation-associated miRNAs, for example, miR-155, are downregulated in CAD patients [86]. On the other hand, likely reflecting cardiac damage, cardiac-specific miRNAs, miR-208a, and miR-133a were upregulated [86]. Following an examination of four possible miRNA transport modalities (microparticles, exosomes, Ago, and HDL transported miRNAs), Finn et al. [88] further noted that especially microparticles of CAD patients generally present lower levels of miRNAs as a result of decreased miRNA loading into microparticles; microparticles from CAD patients were also shown to be less sufficient with respect to miRNA delivery to cultivated cells (probably due to the lowered expression of developmental endothelial locus 1 (Del-1), a known mediator of endothelial microparticle uptake [86]).

Of the above mentioned miRNAs, circulating miR-126 has already been identified as playing a role in intercellular communication [81, 89]. MiR-126 is an endothelial cell enriched miRNA crucial for proper vessel integrity and vascular development [73, 74]; however, its possible involvement in the lipid metabolism was suggested on the basis of a described correlation between miR-126 and LDL circulating levels [90]. During atherosclerosis, endothelial cells undergo both necrosis and apoptosis and miR-126 is subsequently released either in the form of apoptotic bodies [81] or in a complex with the Ago2 protein [89]. Apoptotic bodies were described as being transferred to surrounding endothelial cells protecting them from macrophage infiltration as a result of VCAM1 downregulation [91]. Moreover, miR-126 delivery is inducing CXCL12 expression (through the downregulation of RGS16) which promotes progenitor cell recruitment and healing [81]. On the contrary, Ago2-transferred miR-126 enters vascular smooth muscle cells where it promotes their turnover, thereby promoting the atherosclerotic phenotype [89]. Furthermore, it was shown that monocytic miR-150 is transferred to endothelial cells in the form of microvesicles [75, 76], where it stimulates endothelial cell migration [76] and promotes angiogenesis [75].

The assessment of circulating miRNAs from a biomarker perspective links the downregulation of the above mentioned circulating miR-126 to an increased risk of myocardial infarction [92]. Other potential biomarkers miRNAs include miR-214, whose levels correlate with the severity of CAD [93], and miR-21 and miR-221, whose levels have been shown to be higher and lower, respectively, in patients suffering from atherosclerosis/ischemic stroke [94].

### 3.3. HDL and LDL Transported MicroRNAs

HDL and LDL particle levels (as well as the level of their cholesterol and miRNA content) are commonly affected in dyslipidemia, hyperlipidemia, and metabolic syndrome patients [87]. Vickers et al. demonstrated that both HDL and LDL particles contain miRNAs and that the profiles of HDL miRNAs in healthy controls (with miR-135a*, miR-188-5p, and miR-877 being the most abundant in HDL particles) differ from familial hypercholesterolemia patients (with miR-223, miR-105, and miR-106a occurring most frequently) [53]. Of the above-mentioned miRNAs, it is worth pointing out that miR-223 is the most common miRNA transported within HDL molecules. MiR-223 targets GLUT4, as experimentally validated in the heart, which may provide a connection between circulating lipoprotein content and insulin resistance [95]. Other miR-223 targets include scavenger receptor class B type I (SR-BI) [96], NOD-like receptor pyrin domain containing 3 (NLRP3) [97], and intercellular adhesion molecule 1 (ICAM-1) [98]. SR-BI is crucial in the HDL cholesterol metabolism and its downregulation by miR-223 in human hepatic cells may result in decreased HDL cholesterol uptake, thus affecting reverse cholesterol transport [96]. In addition to its function in reverse cholesterol transport, HDL cholesterol also has anti-inflammatory, antithrombotic, and antioxidative properties [99]. The anti-inflammatory effect of HDL particles may be at least in part mediated by miR-223 targeting of NLRP3, a well-known inflammasome which responds to the various forms of cellular stress [97], and also by targeting ICAM-1 in endothelial cells [98]. Since miR-223 is not commonly transcribed in endothelial cells, its delivery by HDL particles represents a new anti-inflammatory mechanism for the protection of the endothelium against leukocyte infiltration [98]. In the future, miR-223 may potentially be used as a mediator of other HDL functions. More information regarding miR-233 may be obtained from a recent review by Haneklaus et al. [100].

LDL miRNA profiles have been shown to be more similar to exosomal miRNAs content than to HDL miRNA content [53]. A study by Wagner et al. indicated that LDL generally contains less miRNAs than HDL [77], with the sole exception of miR-155 which exhibits higher levels in LDL than in HDL [77]. MiR-155 is a proinflammatory miRNA and its conflicting role in atherosclerosis has only been described recently (see [101] for a review). Despite its conflicting role, the association of LDL with inflammation is a well-accepted fact and miR-155 may thus provide another key to this relationship [77, 101].

It is important to note the existence of studies which show that miRNAs may be transferred from HDL particles to recipient cells [53, 77, 98]. A study by Vickers et al. describes the SR-BI-mediated internalization of HDL particles with subsequent changes in the transcriptional regulation of the recipient cell [53]. Although a subsequent study by Wagner et al. confirmed Vickers' results, in the case of* in vitro* models of endothelial cells, smooth muscle cells, and macrophages, the study indicated that this miRNA uptake is not sufficient due to the low amounts of miRNAs being transferred from HDL to target cells [77]. Interestingly, Wagner et al. also showed that native HDL particles are able to cause transient downregulation of some miRNA in the recipient cells, which is most likely caused by the pumping of miRNAs into the HDL [77]. In a very recent study by Tabet et al., the incubation of endothelial cells with HDL particles caused the significant upregulation of HDL-abundant miR-223, thereby leading to ICAM-1 downregulation [98].

## 4. Therapeutic Potential of Lipid Metabolism miRNAs in Atherosclerosis

Hyperlipidemia is one of the most critical risk factors contributing to atherosclerosis, along with other well-known factors such as smoking, obesity, or insulin resistance. At present, most therapeutic strategies focus on lowering LDL-cholesterol, for example, by using statins, a strategy which has been known to lower patients' mortality [102]. However, a more complex approach, focusing on lowering the levels of circulating fatty acids and increasing the levels of HDL, is now being investigated [43]. Due to their multitargeting essence, miRNAs may thus become very powerful tools, influencing all stages of the pathogenic process of hyperlipidemia/dyslipidemia and positively affecting the entire blood lipid spectrum of affected patients. At present, there are two main ways of using miRNAs in therapy: the inhibition strategy uses antagomiRs (sequences that bind to target miRNA and block its function) while replacement therapy uses miRNA mimics [103].

Most of the work in the field of cardiometabolic diseases has so far focused on miR-33 [104–108]. Indeed, this is one of the miRNAs that Regulus Therapeutics is preparing for future clinical use in the treatment of atherosclerosis [109]. Since the effects of this miRNA are mostly proatherosclerotic, as described above, antagomiR therapy against miR-33 would be a reasonable choice [104–106].

Rodent models indicated that miR-33 affects the development of atherosclerosis; however, the results are still not consistent across all research groups [104–106, 108]. Using LDL-receptor knockout mice (LDR-R^−/−^), the short-term (4 weeks) application of anti-miR-33 led to an increase in HDL concentrations and increased reverse cholesterol transport, consequently leading to atherosclerosis regression [108]. However, two longitudinal studies conducted since provided disparate results: a study by Tyler et al. showed that LDR-R^−/−^ mice treated with anti-miR-33 for 14 weeks only exhibit a slight increase in HDL concentrations during the first two weeks, with no effect on HDL levels taking place during the following weeks. Moreover, levels of circulating triglycerides were higher in anti-miR-33-treated animals at the end of the study and no significant increasing trend with respect to body weight was observed [104]. The most recent study by Rotlan et al. indicated that long-term (12 weeks) application of anti-miR-33 increases HDL concentrations with increased cholesterol efflux capacity, but only in chow diet-fed animals [105]. Moreover, atherosclerotic plaque sizes were significantly reduced, as was the content of infiltrating macrophages [105]. Since there are differences in design of both above-mentioned studies, especially regarding antagomiR chemistry and the diet composition of experimental animals, additional studies are needed to reveal the sources of observed results variability.

Using a slightly different model with ApoB and miR-33 double knockout (ApoB^−/−^ miR-33^−/−^) mice, Horie et al. reported that miR-33 deficiency caused the upregulation of HDL and slowed the progression of atherosclerosis, that is, results similar to the application of anti-miR-33 by Horie et al. [106]. However, since rodents lack one SREBP and consequently one form of miR-33, the extent to which results obtained from rodents may be applied to humans remains somewhat questionable. Experiments employing nonhuman primates (African green monkeys) were therefore performed, leading to the finding that anti-miR-33 therapy not only increased HDL cholesterol levels but also decreased the levels of circulating triglycerides, thus significantly reducing the risk of future atherosclerosis development [107].

With respect to additional miRNAs mentioned in this review, miR-122 also seems to be a promising tool for normalizing the lipid spectrum, especially as the effects of miravirsen—currently the first miRNA-based therapeutic used in the treatment of the hepatitis C virus infection in African green monkeys—indicated positive hypolipidemic effects [110]. The application of miR-126 also seems to be very promising, since the application of apoptotic bodies resulted in the regression of atherosclerotic plaques in experimental animals [81]; however, with respect to its possible proatherogenic effect via vascular smooth muscle cell activation [89], additional experimental work must be carried out prior to its potential clinical utilization. Last but not least, miR-30c, a potential candidate for miRNA mimic therapy, should also be mentioned: MiR-30c is embedded within the intron of* NFYC* (miR-33* target) and has recently been described as affecting the lipid metabolism. The hepatic overexpression of miR-30c decreases lipid synthesis and downregulates circulating lipid levels and vice versa: the inhibition of miR-30c expression using antagomiRs resulted in the acceleration of the atherosclerosis process in ApoE^−/−^ mice [29].

## 5. Conclusion

MicroRNAs have repeatedly been shown to play key roles in various processes within the body, including the lipid metabolism. Since the lipid metabolism regulatory networks are themselves extremely complex, the addition of miRNAs to this intricate network clearly calls for a period of fine-tuning of the established links as well as a time for embarking on a search for new connections. Since one miRNA usually targets a group of genes and one gene may be targeted by a group of miRNAs with other miRNAs possibly embedded within corresponding introns, we might conclude by saying that the lipid metabolism is controlled by complex miRNAs-mRNAs-miRNAs networks. Nevertheless, understanding these complicated regulatory networks is only a matter of time and effort, both of which are necessary for the development of effective therapies for atherosclerosis as well as for the identification of new biomarkers which will not only reflect the severity of the disease but also predict its progression.

## Figures and Tables

**Table 1:** Overview of miR-33, miR-122, miR-27a, miR-27b, miR-144, and miR-370 functions.

MicroRNA	Target	Function	Model	Reference
miR-33/miR-33*	ABCA1	Cholesterol loading into HDL particles	THP-1, HepG2, J774, HEPA, Fu5AH, EAhy296, COS-7, and 293T cells; C57BL/6 and Ldlr^−/−^mice [6] HEK293, J774, HepG2, and IMR-90 cells, C57BL/6J mice [7] HepG2, THP1, and Y1 cells; C57/BL6 mice [8] Huh7, THP1, COS7 cells; C57BL/6 mice; rhesus monkey (*Macaca mulatta*) [9]	[6–10]

miR33/miR-33*	NPC1	Cholesterol transport within cell from the lysosomal compartment	Huh7, THP1, COS7 cells; C57BL/6 mice; rhesus monkey (*Macaca mulatta*) [9]	[9]

miR-33	ABCG1	HDL formation and reverse cholesterol transport	THP-1, HepG2, J774, HEPA, Fu5AH, EAhy296, COS-7 and 293T cells; C57BL/6 and Ldlr^−/−^ mice [6] HEK293, Hep3B cells; Human monocyte-derived macrophages; C57BL/6J mice [10]	[6, 10]

miR-33	ABCB11, ATP8B1	Cholesterol transport from hepatocyte to biliary ducts (cholesterol retention)	HEK293 and HuH-7 cells, C57BL/6 mice; Isolated mouse hepatocytes	[11]

miR-33/miR-33*	CPT1A, CROT	Fatty acid oxidation (upregulation of miR-33/33* leads to inhibition of fatty acids oxidation)	HepG2, THP1 and Y1 cells; C57/BL6 mice [8] Huh7, THP1, COS7 cells; C57BL/6 mice; rhesus monkey (*Macaca mulatta*) [9]	[8, 9]

miR-33	HADBH, SIRT6, AMPK*α*	Fatty acid oxidation (upregulation of miR-33 leads to inhibition of fatty acids oxidation)	HepG2, THP1 and Y1 cells; C57/BL6 mice	[8]

miR-33*	SRC1, SRC3, NFYC, RIP140	Fatty acid oxidation (upregulation of miR-33* leads to inhibition of fatty acids oxidation)	Huh7, THP1, COS7 cells; C57BL/6 mice; rhesus monkey (*Macaca mulatta*) [9]	[9]

miR-33	IRS-2, G6PC, PCK1	Insulin signaling and glucose metabolism	Huh7, THP1, COS7 cells; C57BL/6 mice; rhesus monkey (*Macaca mulatta*) [9]	[9]

miR-122	—	Anti-miR-122 introduction leads to decrease in plasmatic cholesterol	C57BL/6 mice [12] Primary hepatocytes from Balb/c mice, C57BL/6 mice [13]	[12, 13]

miR-122	SREBP and other targets	Anti-miR-122 delivery changes expression of a huge number of genes, including *SREBP*. This results in increase in fatty acid oxidation and decreases fatty acid and cholesterol synthesis. This results in the improvement of liver steatosis.	Primary hepatocytes from Balb/c mice, C57BL/6 mice	[13]

miR-122	MTTP, Klf6	Knockdown animals present with lower levels of circulating cholesterol and fatty acids. However, lipids accumulate in the livers (MTTP) of experimental animals leading to hepatosteatosis, fibrosis (Klf6), and tumor formation.	Mir122 conditional knockout (Mir122^loxP/loxP^) mice [14] Mir122a^−/−^ mice; computational prediction [15]	[14, 15]

miR-27a	RXR*α*, ABCA1, FASN, SREBP1, SREBP2, PPAR*α*, PPAR*γ* ApoA1, ApoB100, ApoE3		Huh-7.5 cells [16] HuH7, HepG2, HEK29 and HeLa cells [17]	[16, 17]

miR-27b	PPAR*γ*, ANGPTL3, NDST1, *GPAM *	miR-27b is predicted to target 27 mRNAs involved in lipid metabolism; targets in the second column have already been validated.	C57BL/6J mice, Huh7 cells, computational prediction [18]	[18]

miR-27b	PPAR*γ*, C/EBP*α*	Downregulation of PPAR*γ* and C/EBP*α* by miR-27b leads to blockade in adipocyte differentiation.	3T3-L1, OP9 and C2C12 cells [19] 3T3-L1 cells, C57BL/6J mice [20]	[19, 20]

miR-27b	PPAR*α*	Targeting PPAR*α* with miR-27b affects indirectly the expression of ABCA1 and ABCG1 (PPAR*α* targets).	3T3-L1 cells, C57BL/6J mice [20] HuH7, HepG2, HEK29 and HeLa cells [17]	[17, 20]

miR-370	CPT1A	MiR-370 affects miR-122 expression and directly targets CPT1A thus affecting fatty acid oxidation.	C57BL/6 and apoE^−/−^ mice [21]	[21]

miR-144	ABCA1	miR-144 expression is regulated by LXR and FXR. MiR-144 itself targets ABCA1 thus affecting cholesterol metabolism.	C57BL/6J mice [22] J774, THP-1, HepG2, Huh-7, Hepa, and EAhy926 cells; C57BL/6 mice [23]	[22, 23]

ABCA1: ATP-binding cassette A1; NPC1: Niemann-Pick disease C1; ABCG1: ATP-binding cassette G1; ABCB11: ATP-binding cassette B11; ATP8B1: ATPase class I type 8B member 1; CPT1A: carnitine palmitoyltransferase 1A; CROT: carnitine O-octaniltransferase; HADBH: hydroxyacyl-CoA-dehydrogenase; SIRT6: sirtuin-6; AMPK*α*: AMP-activated protein kinase subunit-*α*; SRC1: steroid receptor coactivator 1; SRC3: steroid receptor coactivator 3; NFYC: nuclear transcription factor Y; RIP140: receptor-interacting protein 140; IRS-2: insulin receptor substrate 2; G6PC: glucose-6-phosphatase; PCK1: phosphoenolpyruvate carboxykinase; SREBP: sterol regulatory element-binding protein; MTTP: microsomal triglyceride transfer protein; Klf6: Kruppel-like factor 6; RXR*α*: retinoid X receptor *α*; FASN: fatty acid synthase; PPAR*α*: peroxisome proliferator-activated receptor *α*; PPAR*γ*: peroxisome proliferator-activated receptor *γ*; ApoA1: apolipoprotein A1; ApoB100: apolipoprotein B100; ApoE3: apolipoprotein E3; ANGPTL3: angiopoietin-like 3; NDST1: N-deacetylase-N-sulfotransferase 1; GPAM: glycerol-3-phosphate acyltransferase; C/EBP*α*: CCAAT/enhancer-binding protein *α*.

**Table 2:** Additional microRNAs involved in the lipid metabolism and atherosclerosis.

MicroRNA	Target mRNA	Function	Reference
miR-1/206	LXR*α*	Suppresses lipogenesis.	[24]

miR-9	ACAT1	Decreases formation of foam cells.	[25]
	PPAR*δ*	Mediates inflammatory response in human monocytes following lipopolysaccharide treatment.	[26]

miR-21	FABP7	Downregulated in the livers of mice on a high-fat diet. MiR-21 levels may be upregulated by lycopene, thus blocking lipid accumulation.	[27]

miR-29a	LPL	Upregulated in oxLDL-treated dendritic cells. By targeting LPL, it affects lipid uptake and inflammatory cytokine secretion.	[28]

miR-30c	MTP	Reduces ApoB secretion, lipid synthesis, and atherosclerosis in ApoE^−/−^ mice.	[29]

miR-125a-5p	ORP9	Upregulated in macrophages treated with oxLDL mediating lipid uptake. Inhibits the secretion of inflammatory cytokines (IL-2, IL-6-, TNF*α*, TGF*β*).	[30]

miR-155	SCG2	Upregulated in oxLDL-treated macrophages/dendritic cells. Important for lipid uptake and expression of adhesion molecules.	[31]
	LXR*α*	Upregulated in the liver of animals on a high-fat diet. Upregulation seems to protect them from steatosis, since miR-155^−/−^ animals are susceptible to hepatosteatosis.	[32]
	FADD	Attenuates oxLDL-mediated macrophage apoptosis implicating a possible protective role in atherosclerosis.	[33]

miR-181b	Importin-*α*3	Downregulated in ApoE^−/−^ mice and in subjects with CAD. Delivery of miR-181b reduces inflammatory response and protects ApoE^−/−^ mice from atherosclerosis.	[34]

miR-185/342	SREBP	In prostate cancer cells, miR-185 and miR-342 target SREBP, causing FASN and HMGCoAR downregulation.	[35]

miR-217	SIRT1	Upregulated in the liver of chronically ethanol-fed mice, resulting in fat accumulation.	[36]

miR-335	—	Upregulated in the liver and white adipose tissue of obese mice. During adipose tissue differentiation, its levels correlate with lipid accumulation and e.g., PPAR*γ* or FAS levels.	[37]

miR-378/378*	CRAT, MED13	Both miRNAs are encoded within the PGC-1*β* gene which is important for mitochondrial development and metabolism. By targeting CRAT and MED13, they affect fatty acid metabolism and metabolic gene expression control.	[38]
	—	Overexpression of miR-378/378* stimulates lipogenesis and increases lipid droplet size in developing adipocytes.	[39]

miR-467b	LPL	Downregulated in the liver of high-fat diet-fed mice, which results in the upregulation of LPL and affects the development of hepatosteatosis.	[40]
		Decreased lipid accumulation and inflammatory cytokine secretion in oxLDL-treated macrophages.	[41]

miR-613	LXR*α*	Suppresses lipogenesis.	[42]

LXR*α*: liver X receptor; ACAT1: acyl-coenzyme A: cholesterol acyltransferase; PPAR*γ*: peroxisome proliferator-activated receptor *γ*; FABP7: fatty acid-binding protein 7; LPL: lipoprotein lipase; MTP: microsomal triglyceride transfer protein; ORP9: oxysterol binding protein-like 9; SCG2: secretogranin 2; FADD: fas-associated death domain-containing protein; SREBP: sterol regulatory element binding protein; SIRT1: sirtuin 1; CRAT: carnitine O-acetyltransferase; MED13: mediator of RNA polymerase II transcription subunit 13; oxLDL: oxidized low-density lipoprotein.
